# TFG-β Nuclear Staining as a Potential Relapse Risk Factor in Early-Stage Non-Small-Cell Lung Cancer

**DOI:** 10.3390/ijms232213780

**Published:** 2022-11-09

**Authors:** Nuria Cárdenas-Quesada, Leticia Díaz-Beltrán, Carmen Rosa-Garrido, Bélgica Márquez-Lobo, Adela Sabio-González, Rafael J. Luque-Barona, María Isabel Núñez, Pedro Sánchez-Rovira

**Affiliations:** 1 Medical Oncology Unit, University Hospital of Jaén, 23007 Jaén, Spain; 2 Andalusian Public Foundation for Biosanitary Research in Eastern Andalusia (FIBAO), University Hospital of Jaén, 23007 Jaén, Spain; 3 Provincial Intercentre Unit of Pathological Anatomy of the Province of Granada (UPIGAP), Clínico San Cecilio University Hospital, 18016 Granada, Spain; 4 Pathological Anatomy Unit, University Hospital of Jaén, 23007 Jaén, Spain; 5 Department of Radiology and Physical Medicine, Granada University, 18016 Granada, Spain; 6 Biopathology and Regenerative Medicine Institute (IBIMER), Center for Biomedical Research (CIBM), University of Granada, 18100 Granada, Spain; 7 Biosanitary Research Institute, ibs.Granada, 18016 Granada, Spain

**Keywords:** PD-L1, TILs, TGF-β, prognostic factors, checkpoint inhibition, early-stage lung cancer

## Abstract

Nowadays, the impact of the tumor-immune microenvironment (TME) in non-small-cell lung cancer (NSCLC) prognosis and treatment response remains unclear. Thus, we evaluated the expression of PD-L1, tumor-infiltrating lymphocytes (TILs), and transforming growth factor beta (TGF-β) in NSCLC to identify differences in TME, detect possible new prognostic factors, and assess their relationship. We retrospectively analyzed 55 samples from patients who underwent NSCLC surgery and had over a 5-year follow-up. PD-L1 expression was determined by immunohistochemistry following standard techniques. The presence of TILs was evaluated at low magnification and classified into two categories, “intense” and “non-intense”. Cytoplasmic TGF-β staining visualization was divided into four categories, and unequivocal nuclear staining in >1% of viable tumor cells was defined as “present” or “absent”. Our aim was to identify differences in disease-free survival (DFS) and overall survival (OS). Tumor stage was the only objective prognostic factor for OS. PD-L1 expression and the presence of TILs had no prognostic impact, neither their combination. There seems to be a lower expression of PD-L1 and a higher expression of TILs in early stages of the disease. Our TGF-β nuclear staining analysis was promising, since it was associated with worse DFS, revealing this protein as a possible prognostic biomarker of recurrence for resectable NSCLC.

## 1. Introduction

Lung cancer (LC) is a devastating disease and a major therapeutic burden with poor survival rates. Nowadays, it is the second most common tumor worldwide and is responsible for the highest number of cancer deaths [1]. Despite current evidence [2,3,4], screening tests have not yet been standardised in routine clinical practice to improve early diagnosis. In fact, almost 80% of cases are still diagnosed at advanced, inoperable stages.

Recently, the identification of comprehensive lung cancer molecular profiles has improved the understanding of this pathology, which has become the paradigm of molecular oncology. Thus, the discovery of driver genetic alterations and potential molecular targets has led towards a better staging and diagnosis of the disease, allowing the development of personalized treatments for an increasing number of patients [5,6,7,8,9]. In the early stages of NSCLC (stage I and II), surgery remains the standard treatment in clinically operable patients [10], with a 5-year OS ranging from 92% (stage IA1) to 41% (stage IIIA) [6]. Adjuvant chemotherapy (CT) maintains its benefit for patients with N1 and N2 disease (stages II and III), with an overall improvement in absolute survival of 4% to 5% at 5 years [11]. The studies on neoadjuvant CT are less comprehensive, and the results comparison of both therapies did not show a significant difference in OS [12].

These poor survival rates make necessary the identification of new biomarkers that can be used as prognostic factors for lung cancer (neo)adjuvant targeted therapies [13]. In this line, Osimertinib has recently been approved for use as adjuvant therapy after complete tumor resection in patients with stage IB-IIIA NSCLC with epidermal growth factor receptor (EGFR) exon 19 deletions or exon 21 L858R mutations [14].

Prior research in the field of advanced NSCLCs led to an expanded reach and impact of immune checkpoint inhibitors (ICIs), a new therapeutic setting as part of a frontline treatment strategy. Similarly, their use along with CT in neoadjuvant IB-IIIA NSCLC has also shown an increased pathological complete response rate compared to CT alone [15]. Likewise, adjuvant ICIs with anti-PD-L1 has led to increased DFS when compared to best supportive care for patients with PD-L1-positive tumors [16]. Additionally, a phase II clinical trial supports the addition of neoadjuvant Nivolumab to platinum-based CT in patients with resectable stage IIIA NSCLC [17].

So far, however, conflicting data have been obtained about the prognostic and predictive power of the newly discovered biomarkers and treatments under study [17,18,19,20]. In this sense, strict, precise, and sensitive biomarkers are still lacking in ICIs treatment to select responders, as is the case with targeted therapies. Hence, there continues to be much interest and, indeed, much progress in elucidating molecular targets that assist with prognosis assessment and treatment selection for patients with operated early-stage NSCLC, with the future moving towards precision medicine and individualised patient management. Ultimately, the development of affordable and reproducible biomarkers has become essential to predict the adjuvant therapeutic efficacy and recurrence rate for these patients. In this sense, nuclear TGF-β staining meets these criteria and arises as a potential molecular target whose expression may significantly predict a worse prognosis for resectable lung cancer patients at its early stage [21]. In addition, TGF-β inhibition has an impact on the production of regulatory T cells and may enhance the effect of PD-1/PD-L1 inhibitors, leading to improved responses to these therapies [22].

Here, we aimed to evaluate the association of clinicopathologic characteristics and the prognostic value of PDL-1 expression and TILs, and explored the immune microenvironment by assessing their relationship, since they have been suggested as clinically applicable predictive biomarkers in surgically resectable NSCLC. Furthermore, we studied TGF-β expression and proposed a more simple and efficient way for its assessment (nuclear staining), since this molecular target has been outlined as a potential prognostic biomarker that may open a new line of research pointed towards TGF-β inhibitor therapies in NSCLC, in combination with ICIs in those patients with a worse prognosis.

The chi-squared test, Fisher’s exact test, and the student’s *t*-test were used to study the differences in patient characteristics according to their results in the previous biomarker analyses of PD-L1, TILs, and TGF-beta expression. The normality study of the numerical variables was carried out using the Shapiro–Wilk test. The evaluation of the differences in survival curves, obtained by Kaplan–Meier, was carried out using the log-rank test. Cox regression was applied to obtain the crude hazard ratio (HRc) of the study variables and the adjusted hazard ratio (HRa) according to the stage, sex, and age of the patients. We used a significance level of 5%. Statistical analyses were carried out with IBM SPSS V21 and R version 4.0.3.

## 2. Results

### 2.1. Baseline Patient and Tumor Characteristics

A comparative analysis between patients with an analyzable sample (n = 55) and those without (n = 39) found no significant differences in the distribution of the variables that we consider most important in the evolution of the disease: sex, age, squamous vs. non-squamous histology, stage (I, II, III), type of surgery, and administration of CT (Supplementary Table S1).

The characteristics of the patients with a sample available for assessment and a comparison of the distribution according to the variables under study are shown in Table 1. Of the 55 patients with an available tumor sample for our analysis, more than 48% of them had died, 38.18% had relapsed (21 relapses), and 12% (7 patients) had either developed a second lung tumor (3 patients) or another primary cancer.

We determined OS and DFS in our sample using Cox model regression according to the following characteristics: sex, age, squamous vs. non-squamous histology, stage, type of surgery, EGFR mutation, and CT administration. Statistically significant differences were found in OS according to staging. Thus, we confirmed that staging was the only independent prognostic factor in terms of survival, with lower staging meaning a higher survival rate.

### 2.2. PD-L1 Expression

PD-L1 expression analysis was performed qualitatively in two ways: in three blocks <1% (41 patients), between 1–49% (5 patients) and ≥50% (9 patients); or in two blocks, pooling ≥1% overall (14 patients) versus <1%.

**Figure 1:**
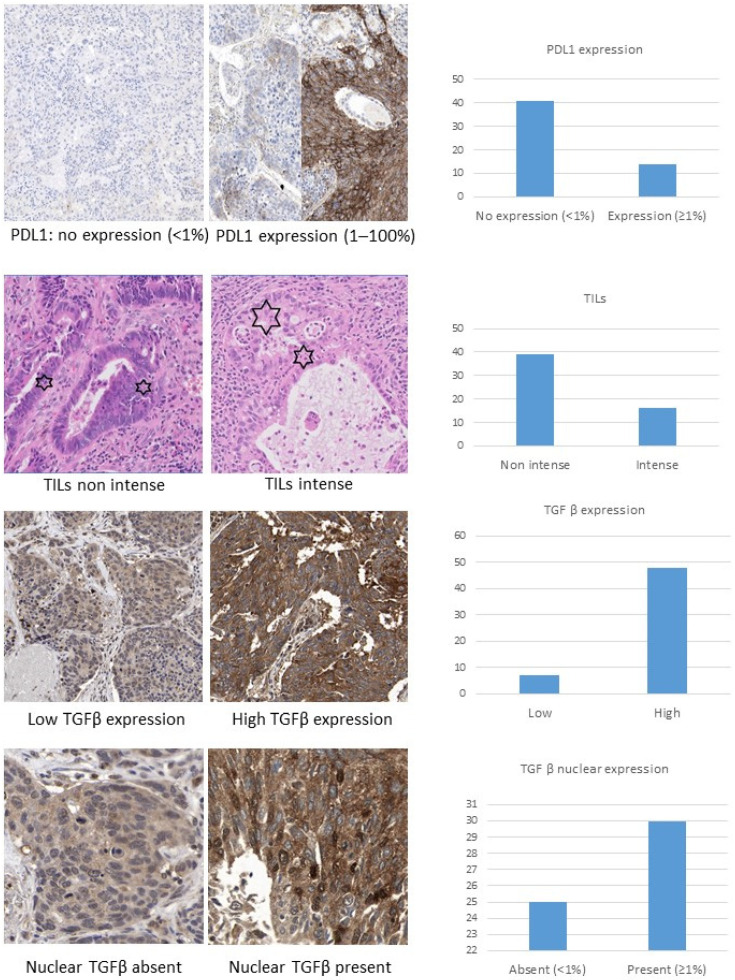
Histological algorithm of evaluation (as described in Section 4.2, Section 4.3 and Section 4.4 of Section 4) and frequency distribution for the biomarkers of interest. The six-pointed stars show intraepithelial lymphocytes. All images at 20× magnification (digital zoom) except for TGF-β nuclear staining (40×).

**Figure 2:**
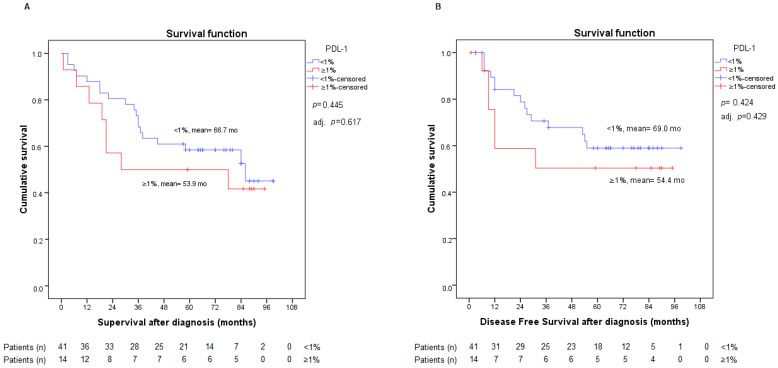
Analysis of overall survival (**A**) and disease-free survival (**B**) according to PD-L1 expression <1% or ≥1%. Adj. *p*: *p*-value from Cox regression model adjusted by stage.

After the analysis of both PD-L1 classifications, we found no statistically significant differences in either OS or DFS. Data of the PD-L1 ≥1% vs. <1% comparison is represented in Figure 1. We considered that these results should be adjusted for the staging variable, as we knew its influence on OS. No differences were found when adjusting for staging. This information is also provided in Figure 2.

We also adjusted PD-L1 expression results for sex and age, and, ultimately, for sex and stage, without obtaining statistically relevant results in either case. Hence, the PD-L1 variable did not appear as a possible risk factor for OS or DFS, even when adjusted for sex, age, and stage.

Likewise, our analysis yielded no relationship among PD-L1 expression and the other two variables studied (TILs and TGF-β), with a *p*-value of 0.306 for TILs, 0.172 for TGF-β, and 1.000 for nuclear staining, respectively.

### 2.3. Presence of TILs

**Figure 3:**
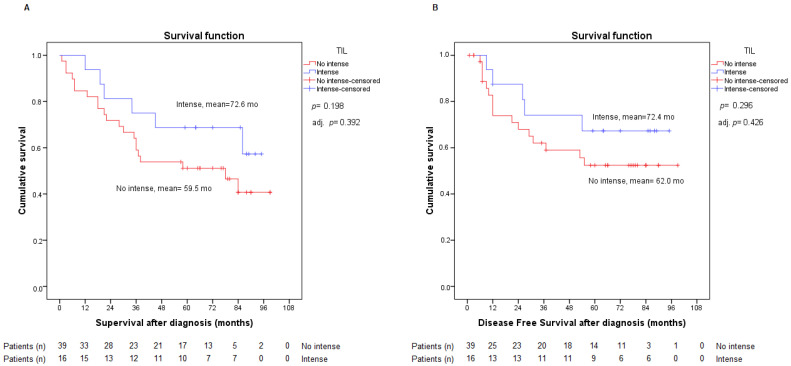
Analysis of overall survival (**A**) and disease-free survival (**B**) according to intense or non-intense TIL expression. Adj. *p*: *p*-value from Cox regression model adjusted by stage.

As previously described, the presence of TILs has been dichotomously assessed (intense, non-intense). In our study, 39 patients presented intense TILs, whereas 16 had non-intense TILs. Our analysis revealed very moderate indications of significance, with a better prognosis in terms of OS with intense TILs (Figure 3). There were no statistically significant differences in DFS between the two groups. Adjusting these data with the staging variable also generated no statistical changes. Adjustments for sex and age, and sex and stage, did not modify our findings either. Therefore, the TILs variable did not manifest as a risk factor for OS and DFS, adjusted for sex, age, and stage.

**Figure 4:**
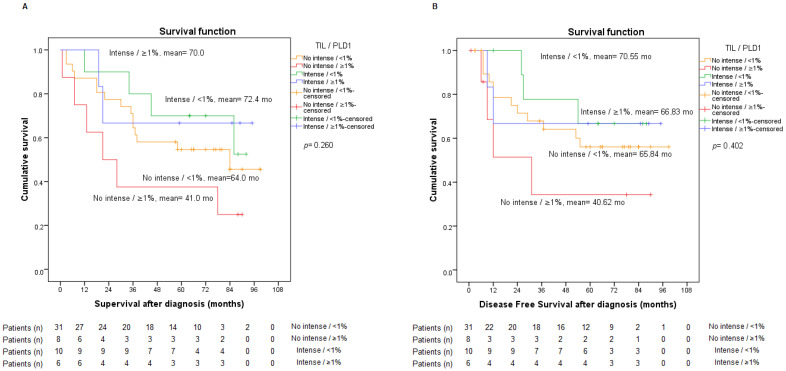
Analysis of overall survival (**A**) and disease-free survival (**B**) according to PD-L1 expression <1% or ≥ 1% and intense or non-intense TIL.

We then decided to evaluate the possible statistical relationship with the other variables (PD-L1 and TGF-β), and as already mentioned above, no relationship between this variable and PD-L1 was found. Our results reported no significant association, with *p*-values of 0.402 regarding TGF-β and 0.645 with respect to nuclear staining. Considering the negative prognostic value of high PD-L1 expression and the positive prognostic value of intense TILs, we analyzed both variables together without finding statistically relevant data, but we did discover that our sample complied with these trends (Figure 4).

### 2.4. Assessment of TGF-β Expression

Regarding TGF- β expression, we classified our patients into two categories: those with low TGF-β expression (n = 7) and those with high expression (n = 48); no patient was tested as negative. They were also divided in two groups according to the absence (n = 25) or presence (n = 30) of TGF-β nuclear staining.

**Figure 5:**
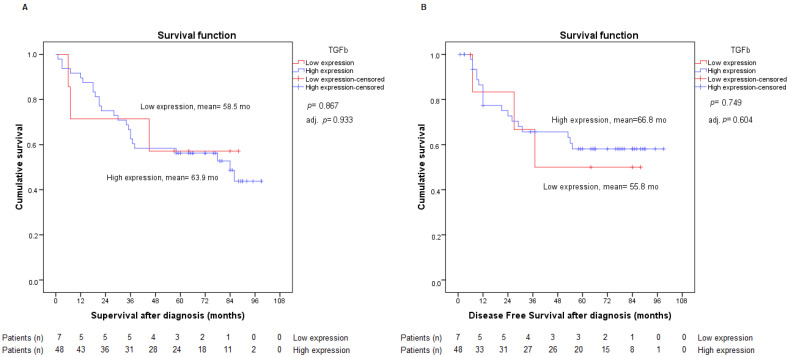
Analysis of overall survival (**A**) and disease-free survival (**B**) according to low or high TGF-β expression. Adj. *p*: *p*-value from Cox regression model adjusted by stage.

In our statistical analysis, no significant differences were found among all TGF-β patients unified in low or high expression (1 vs. 2–3). Figure 5 shows these data and their adjustment for staging, as in the previous variables.

**Figure 6:**
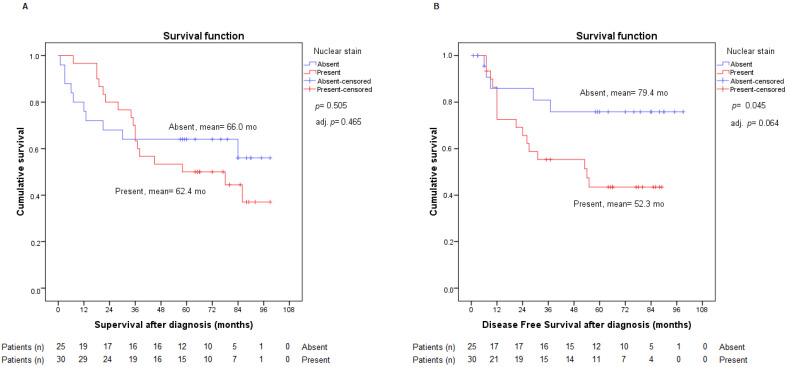
Analysis of overall survival (**A**) and disease-free survival (**B**) according to absent or present TGF-β nuclear staining. Adj. *p*: *p*-value from Cox regression model adjusted by stage.

When analyzing TGF-β nuclear staining, according to the dichotomous variable absent and present, we found statistical significance in the DFS curves in favor of absent nuclear staining, with a *p*-value of 0.045. This outcome was not observed in OS; however, significance was maintained when adjusting the result with the variables age and sex (*p*-value: 0.044, HRa: 2.832, IC HRa: (1.029–7.794)), and it also remained very close to significance if the adjustment was performed by staging (*p*-value 0.064, HRa: 2.597, IC HRa: (0.946–7.126)), as is shown in Figure 6. Therefore, present nuclear staining may arise as a potential relapse risk factor in operated early-stage NSCLC patients, since the risk is more than twice as high for patients with present staining versus patients with absent staining.

**Figure 7:**
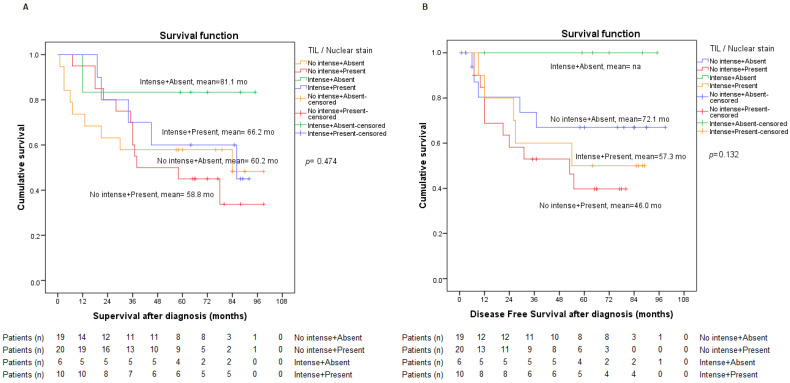
Analysis of overall survival (**A**) and disease-free survival (**B**) according to intense or non-intense TIL and absent or present TGF-β nuclear staining.

Unfortunately, there was no statistically significant association between TGF-β or nuclear staining and the rest of the variables under consideration. The *p*-value obtained between TGF-β and nuclear staining analysis was of 0.226. The analysis of DFS and SG combining TIL and nuclear staining did not show statistically significant data either. The graph, however, is consistent with what has already been reported so far in the literature and described in this study. Thus, patients with intense TIL and no nuclear staining may have a better survival rate, whereas, on the other hand, patients with non-intense TIL and who present nuclear staining could have a worse prognosis (Figure 7).

## 3. Discussion

The success of targeted therapies and new immunotherapy approaches has created a new paradigm of personalized therapy in lung cancer. Thus, the identification of clinically relevant cancer biomarkers as potential new targets for drug development is in critical demand. In this sense, herein we have studied PD-L1 expression, TILs status, and TGF-β expression in early-stage resectable NSCLC patients to assess their value as predictive and prognostic biomarkers.

Although PD-L1 is known to play a major function in suppressing the immune response, its prognostic value is still being discussed and its role in tumor microenvironment has not been fully elucidated yet [23]. Indeed, PD-L1 expression by IHC is particularly controversial as an ICI biomarker due to discordant results in recent research [24]. Despite some studies finding a robust correlation between high PDL1 expression and a worse prognosis [25] and treatment response after concurrent chemotherapy and radiotherapy [26,27], our analysis suggested that high PD-L1 expression has no prognostic significance. This goes in line with recent evidence [19] revealing that PD-L1 expression is influenced by tumor stage, which may limit its use as a predictive and prognostic indicator. The incidence of PD-L1 expression (by 22C3) in patients with resectable NSCLC is relatively lower compared to patients with a more advanced stage. This fact is also consistent with the low percentage of high PD-L1 values observed in our population. On this matter, Tuminello et al. [23] also concluded that patient characteristics, such as gender and stage, may influence both the immune composition of the TME and post-surgical survival, and, therefore, may distort their true relationship. More recently, data analyzed by the GILT group [18] have also confirmed that PD-L1 tumor expression does not seem to affect early-stage lung cancer prognosis. The disparity in results across studies may be explained by the lack of standardization of the diverse analytical techniques available (i.e., use of different antibodies, platforms, and cut-off thresholds) and the inter-observer variation [28].

Regarding other potential biomarkers, TILs have currently gained increasing attention in the treatment and prognosis of NSCLC, as they constitute a local histopathological reflection of the host’s immune response against tumor cells. In this sense, several studies [29,30,31], including ours, analyzed tumor-infiltrating lymphocyte density and its role in cancer progression, revealing that high levels of TILs correlated with longer overall survival and that TILs were more highly expressed in early stages of the disease. Indeed, our analysis showed higher expression of TILs in early-stage operable lung cancer patients and moderate indications of significance with better prognosis in terms of OS with intense TILs scores. It is worth highlighting that TILs expression is not altered by the possible loss of tissue antigenicity, a factor that could influence PD-L1 expression. Overall, these findings may suggest that TILs appear to be a better prognostic marker for survival than PD-L1, at least for early-stage lung cancer. However, our results are in line with recent evidence [18,19,20], not supporting PD-L1 expression, TILs status, or the combination of both as significant prognostic indicators for resectable NSCLC.

Regardless of the evidence, the presence of high TILs highlights the key role that the immune system plays in the tumor evolution and the patient’s outcome. Thus, recent articles are starting to suggest that immune infiltration could be used to determine which patients would benefit most from adjuvant or neoadjuvant immunotherapy [23,24]. For example, in the PIONeeR study [32], patients with advanced NSCLC responding to anti-PD-L1 ICIs (nivolumab, pembrolizumab, or atezolizumab) had a higher percentage of PD-L1-positive tumor cells and a higher infiltration of cytotoxic tumor-infiltrating lymphocytes. In this regard, there are several attempts to classify tumors according to PD-L1 expression and lymphocytic infiltration [33,34] with the consequent therapeutic implications. In fact, the development of an immune score may arise as a novel possible approach to identify patients who would benefit from new immune therapies. Therefore, the evaluation of the immune cell infiltration and PD-L1 expression should be a standard practice in the management of lung cancer patients, despite their limited prognostic relevance.

In this context, TGF-β expression has emerged as a possible mechanism triggering the different responses to ICIs. Thus, TGF-β inhibition affects the production of regulatory T cells and may potentially increase the effect of PD-1/PD-L1 inhibitors. Indeed, clinical trials have demonstrated the safety and activity of therapeutic approaches simultaneously targeting the PD-1/PD-L1 and TGF-β pathways, and the first promising data in this regard have already been published [35]. This evidence suggested the potential significance of TGF-β expression in lung cancer and, therefore, its possible use as a prognostic and predictive biomarker for this condition. Furthermore, TGF-β plays an important role in the alteration of early epithelial cancer cells to invasive metastatic cancer cells by promoting epithelial-to-mesenchymal transition (EMT) [36], and it has recently been stated that its high expression could significantly predict poor prognosis in patients with NSCLC, since it also stimulates angiogenesis and induces immunosuppression [21].

Herein, we aimed to assess the prognostic value of TGF-β in our sample population by dividing its expression values into two groups (low expression and high expression), as performed in Xue et al. 2011 [37] and Huang et al. 2014 [38], without finding significant differences, as previously explained. We, therefore, set out to improve the methodology for assessing TGF-β expression by using the immunohistochemical assessment of nuclear staining in a similar way to other markers in other pathologies [39,40], since, to our knowledge, it had not yet been applied to evaluate TGF-β prognostic relevance in NSCLC. Hence, a second analysis was conducted by assessing nuclear staining to define TGF-β expression in tumor cells. When considering the presence of unequivocal nuclear staining in >1% of viable tumor cells (present or absent) as an additional possible risk factor, the DSF curves revealed clear significance in favor of absent nuclear staining; that is, patients presenting TGF-β nuclear staining may be more likely to relapse.

As a result of its tumor-promoting abilities, TGF-β and its signaling pathway offer potential opportunities for targeted therapy. As mentioned above, several agents targeting various components of this pathway have been studied or are being developed and evaluated in clinical trials [22,35,36,41]. However, little is known about the expression of TGF-β in general and in NSCLC in particular.

Our findings seem promising and may open a new line of research. We recognize, however, that our work has two main limitations. First, this is a retrospective study with a small sample size, so our results should be confirmed with further analysis in a larger cohort. Second, the interpretation of our findings may be constrained, since previous studies evaluating the prognostic impact of TGF-β in NSCLC usually refer to plasma levels of TGF-β, whereas those that are investigating its possible therapeutic activity with inhibitors do not reflect data on TGF-β expression in treated patients.

Notwithstanding, this work proposes a novel methodological approach for the assessment of TGF-β nuclear staining as a possible risk factor for recurrence in resectable NSCLC, which may have a prognostic and predictive impact for this condition. Therefore, if this TGF-β assessment’s techniques were validated and its relationship with relapse in interventional NSCLC were confirmed in other studies, we could conclude that patients presenting this biomarker in their viable tumor cells would benefit from some type of TGF-β inhibitor therapy in combination with ICIs. This would lead us to the improvement of disease-free survival rates, and, consequently, may increase the quality of life for lung cancer patients.

## 4. Materials and Methods

### 4.1. Patients

From the 94 patients who underwent surgery for pathological stages I to IIIA NSCLC at the University Hospital of Jaén between 2010 and 2013, 55 samples available for analysis were included in this retrospective study. Tumor characteristics, histological subtype, differentiation, size, invasive depth, and lymph node metastatic status were assessed by specialized pathologists. We used the seventh edition of the International Association for the Study of Lung Cancer (IASLC), which was current at the date of patient diagnosis. The median follow-up time from diagnosis was 5.0 (0–8.0) years. Tissue collection and analytical methods were performed in accordance with the Declaration of Helsinki and were approved by the Provincial Research Ethics Committee of Jaén.

### 4.2. Assessment of PD-L1 Expression

We performed immunohistochemistry (IHC) analysis (Santa Clara, CA, USA, Dako Autostainer^®^ Link 48) on 3 µm sections using Dako clone 22C3. The tumor proportion score (TPS) described in the PD-L1 IHC 22C3 pharmDx-NSCLC PD-L1 interpretation manual (accessed June 2021) [42] was used to assess PD-L1 expression. TPS is the percentage of viable tumor cells showing any intensity of partial or complete membrane staining (≥1+) with respect to the total number of viable tumor cells present in the sample. We considered PD-L1 expression to be present if TPS ≥ 1%, and a TPS of ≥ 50% was defined as high PD-L1 expression. This interpretation was carried out by two independent specialist pathologists. Photos of both situations can be found in Figure 1.

### 4.3. Assessment of Tumor-Infiltrating Lymphocytes (TILs)

The presence of tumor-infiltrating lymphocytes (TILs) was analyzed under a light microscope by selecting the representative tumor infiltrating area stained with hematoxylin and eosin, and in which there were sufficient viable tumor cells without necrosis. We assessed the density of intratumorally lymphocytic infiltration at low magnification (10×) and classified it into two categories, “intense” and “non-intense”. High intensity was defined as a strong infiltration equivalent to the density seen in a metastatic lymph node, such as that described by Brambilla et al. [29]. This classification was performed by two independent pathologists, with discordant cases being agreed in a second step. The lymphocytic infiltrates observed in our samples were peritumoral and we did not distinguish between lymphocytic cell types. Photos of intense and non-intense TILs are shown in Figure 1.

### 4.4. Assessment of TGF-β Expression

Sections (4 mm) of formalin-fixed-paraffin-embedded NSCLC biopsies were treated for deparaffinization, rehydration, and antigen retrieval using standard procedures (EnVision FLEX reagents, Agilent, Dako). Antibodies against TGF-beta (Abcam 190503/1:50 dilution) were used for immunostaining performed on an automated system (Autostainer link 48, Dako). After antigen retrieval, the slides obtained were independently examined by two pulmonary pathologists using light microscopy.

Two assessments of TGF-beta expression in tumor cells were performed, given the absence of a standardized method for this procedure. First, a semi-quantitatively immunohistochemical assessment was conducted, as previously described in Xue et al. 2011 [37] and Huang et al. 2014 [38]. Taking into account the intensity of staining and the number of positive cells, a classification into four categories was established: negative (−): complete absence of staining; weak (+): weak staining—regardless of the percentage of positive cells—or when moderate staining is observed in ≤30% of the cells; moderate (++): moderate staining is identified in >30% of the cells or intense staining in ≤50%; intense (+++): intense staining in >50% of the cells (Figure 1). Secondly, nuclear staining was assessed as an additional factor, since other studies on pancreatic adenocarcinoma, such as the one by Javle et al. 2014 [39], considered nuclear staining as a criterion for defining TGF-beta expression in tumor cells in a manner analogous to the interpretation of other biomarkers, such as the anti-IDH1-R132H antibody in glial tumors [40]. Thus, we performed the TGF-beta nuclear staining analysis, considering its unequivocal presence in more than 1% of viable cancer cells as tumor TGF-beta positivity (Figure 1).

### 4.5. Statistical Analysis

Quantitative variables were represented by mean and standard deviation, whereas qualitative variables were defined by frequency and percentage (see Table 1 and Supplementary Table S1).

## 5. Conclusions

To date, it remains challenging to delay or avoid tumor relapse in patients with NSCLC. Although cancer immunotherapy with immune checkpoint inhibitors has revolutionized the treatment of this condition, we still lack accurate prognostic and predictive biomarkers to help in clinical decision-making. Our preliminary results suggested that TGF-beta staining may predict poor prognosis in operable early-stage NSCLC patients, even though further research should be conducted to confirm these findings. Thus, the incorporation of TGF-β nuclear staining as a potential prognostic factor of resectable lung cancer should be studied in future work to confirm whether it may represent a viable powerful tool for the identification of patients with a high risk of recurrence being priority candidates for adjuvant treatment with ICIs and TGF-β inhibitors.

## Figures and Tables

**Table 1:** Characteristics of the patients with available sample, and comparison of the distribution according to the study markers.

	Total (n = 55)	PD-L1			TIL			TGF-β			TGF-Β Nuclear Staining		
		<1% (n = 41)	≥1% (n = 14)	*p*-Value	Non-Intense (n = 39)	Intense (n = 16)	*p*-Value	Weak Expression (n = 7)	High Expression (n = 48)	*p*-Value	Absent (n = 25)	Present (n = 30)	*p*-Value
Sex, n (%)				1.000			0.165			0.577			1.000
Male	49 (89.1)	36 (87.8)	13 (92.9)		33 (84.6)	16 (100)		6 (85.7)	43 (89.6)		22 (88.0)	27 (90.0)	
Female	6 (10.9)	5 (12.2)	1 (7.1)		6 (15.4)	0 (0)		1 (14.3)	5 (10.4)		3 (12.0)	3 (10.0)	
Age, mean (SD)	63.93 (9.74)	64.76 (10.17)	61.50 (8.19)	0.284	64.08 (9.73)	63.56 (10.07)	0.861	67.57 (11.23)	63.40 (9.52)	0.294	65.48 (10.19)	62.63 (9.32)	0.285
Histology, n (%)				0.097			0.626			0.686			0.095
squamous cell carcinoma	23 (41.8)	14 (34.1)	9 (64.3)		15 (38.5)	8 (50.0)		2 (28.6)	21 (43.8)		14 (56.0)	9 (30.0)	
non-squamous	32 (58.2)	27 (65.9)	5 (35.7)		24 (61.5)	8 (50.0)		5 (71.4)	27 (56.3)		11 (44.0)	21 (70.0)	
Staging, n (%)				0.189			0.585			1.000			0.864
I	33 (60.0)	27 (65.9)	6 (42.9)		22 (56.4)	11 (68.8)		4 (57.1)	29 (60.4)		14 (56.0)	19 (63.3)	
II	14 (25.5)	10 (24.4)	4 (28.6)		10 (25.6)	4 (25.0)		2 (28.6)	12 (25.0)		7 (28.0)	7 (23.3)	
III	8 (14.5)	4 (9.8)	4 (28.6)		7 (17.9)	1 (6.3)		1 (14.3)	7 (14.6)		4 (16.0)	4 (13.3)	
Type of surgery, n (%)				0.099			0.698			0.211			0.542
Limited resection (segmentectomy or wedge resection)	4 (7.3)	4 (9.8)	0 (0)		3 (7.7)	1 (6.3)		1 (14.3)	3 (6.3)		3 (12.0)	1 (3.3)	
Lobectomy	39 (70.9)	31 (75.6)	8 (57.1)		26 (66.7)	13 (81.3)		6 (85.7)	33 (68.8)		17 (68.0)	22 (73.3)	
Pneumonectomy	12 (21.8)	6 (14.6)	6 (42.9)		10 (25.6)	2 (12.5)		0 (0)	12 (25.0)		5 (20.0)	7 (23.3)	
EGFR mutation, n (%)				0.742			0.343			0.761			0.039
Positive	2 (3.6)	2 (4.9)	0 (0)		1 (2.6)	1 (6.3)		0 (0)	2 (4.2)		0 (0)	2 (6.7)	
wt	31 (56.4)	24 (58.5)	7 (50.0)		24 (61.5)	7 (43.8)		5 (71.4)	26 (54.2)		11 (44.0)	20 (66.7)	
Unknown	22 (40.0)	15 (36.6)	7 (50.0)		14 (35.9)	8 (50.0)		2 (28.6)	20 (41.7)		14 (56.0)	8 (26.7)	
Chemotherapy, n (%)				0.099			0.527			0.346			0.541
No	33 (60.0)	27 (65.9)	6 (42.9)		25 (64.1)	8 (50.0)		4 (57.1)	29 (60.4)		17 (68.0)	16 (53.3)	
Adjuvant	15 (27.3)	8 (19.5)	7 (50.0)		10 (25.6)	5 (31.3)		1 (14.3)	14 (29.2)		(24.0)	9 (30.0)	
Neo Adjuvant	7 (12.7)	6 (14.6)	1 (7.1)		4 (10.3)	3 (18.8)		2 (28.6)	5 (10.4)		2 (8.0)	5 (16.7)	

Categorical variables are represented by frequency and percentage. Age is represented by the mean and standard deviation (SD). The *p* values are calculated through the *t*-Student test, chi-squared test, or Fisher’s exact test.

## Data Availability

Not applicable.

## Supplementary Materials

The supporting information can be downloaded at: https://www.mdpi.com/article/10.3390/ijms232213780/s1.
