# Supplementary material for: TFG-β Nuclear Staining as a Potential Relapse Risk Factor in Early-Stage Non-Small-Cell Lung Cancer
Source: Int J Mol Sci. 2022 Nov 9;23(22):13780. doi: 10.3390/ijms232213780 (PMC9694009; doi:10.3390/ijms232213780)
Supplement: Supplementary file 1 [file ijms-23-13780-s001.zip › ijms-1955585-supplementary.pdf]

SUPPLEMENTARY MATERIAL

Supplementary Table

Table S1. Comparative study of patients with and without an analyzable sample

	Sample		
	No (n=39)	Yes (n=55)	p-value
**Sex, n (%)**			0.111
Male	29 (74.4)	49 (89.1)	
Female	10 (25.6)	6 (10.9)	
**Age, mean (SD)**	63.62 (10.13)	63.93 (9.74)	0.881
**Histology, n (%)**			1.000
squamous cell carcinoma	14 (42.4)	23 (42.6)	
non-squamous	19 (57.6)	31 (57.4)	
**Staging, n (%)**			0.350
I	19 (50.0)	33 (60.0)	
II	15 (39.5)	14 (25.5)	
III	4 (10.5)	8 (14.5)	
**Type of surgery, n (%)**			0.937
Limited resection (segmentectomy or wedge resection)	3 (7.7)	4 (7.3)	
Lobectomy	29 (74.4)	39 (70,9)	
Pneumonectomy	7 (17.9)	12 (21.8)	
**Chemotherapy, n (%)**			0.230
No	17 (43.6)	33 (60.0)	
Adjuvant	17 (43.6)	15 (27.3)	
Neo Adjuvant	5 (12.8)	7 (12.7)	

Categorical variables are represented by frequency and percentage. Age is represented by the mean and standard deviation (SD). The p-values were calculated using the t-Student test, chi-square test or Fisher's exact test, as appropriate.
